# Long‐term disease control of disseminated superficial actinic porokeratosis (DSAP) with simvastatin 2%/ cholesterol 2% cream

**DOI:** 10.1111/ddg.15950

**Published:** 2025-11-29

**Authors:** Berenice M. Lang, Gregor Ojak, Mandy Crummenauer, Joanna Wegner, Caroline Mann, Stephan Grabbe, Petra Staubach

**Affiliations:** ^1^ Department of Dermatology University medicine Mainz Mainz Germany; ^2^ Department of Dermatology University medicine Frankfurt Frankfurt am Main Germany; ^3^ Dermatology Center Kaiserslautern Germany

**Keywords:** Disease control, porokeratosis, skin cancer prevention, targeted therapy, topical therapy, unmet medical need

## Abstract

**Introduction:**

Disseminated superficial actinic porokeratosis (DSAP) is a rare keratinization disorder associated with an increased risk of cutaneous tumors.

**Objectives:**

To develop a feasible and easy‐to‐apply treatment regimen for long‐time disease control of DSAP including prevention of new skin tumors.

**Patients and Methods:**

19 patients with DSAP were treated with 2% simvastatin/2% cholesterol (SC) cream for a maximum period of 18 months. Disease severity was assessed via *Investigator Global Assessment* (IGA), and quality of life (QoL) was measured using the *Dermatology Life Quality Index* (DLQI). Adverse events and tumor development were documented.

**Results:**

Significant improvement in IGA scores was observed across all treated regions (p < 0.001), with most progress occurring in the first 3 months but maintained under a reduced proactive application schedule twice weekly. DLQI scores decreased significantly within the first 3 months. No new skin tumors developed during treatment.

**Conclusions:**

SC cream is a promising long‐term, targeted treatment for DSAP, offering sustained efficacy, good tolerability, and improved QoL. The individual tapering from daily use to a proactive approach twice a week may be a key factor for disease control and may become an important component of skin tumor prevention in this specific patient population.

## INTRODUCTION

Disseminated superficial actinic porokeratosis (DSAP) is a rare keratinization disorder of the skin, characterized by both genetic and clinical heterogeneity. It primarily affects sun‐exposed areas and typically presents as asymptomatic or pruritic pink to brown papules or plaques, surrounded by a distinctive raised border known as a cornoid lamella. Precise epidemiological data are scarce, largely due to underreporting and frequent misdiagnosis. The condition usually manifests between the fourth and fifth decade of life, with a slight female predominance. Both autosomal‐dominant and sporadic cases have been reported. DSAP predominantly occurs in individuals with fair skin and a history of chronic sun exposure. Beyond its cosmetic and symptomatic burden, DSAP is associated with an elevated risk of squamous cell carcinoma, emphasizing the importance of effective long‐term disease management. Different sources quote a lifetime transformation risk of 3% to 11%, with elevated rates in longer disease progression. Current therapeutic options – such as photodynamic therapy, topical imiquimod, fluorouracil, and retinoids – often yield limited success and are frequently accompanied by adverse effects, including irritation, erythema, and discomfort. Notably, no approved on‐label treatment exists, underscoring the significant unmet medical need for more effective and targeted therapies.^1^, ^2^

Recent advances in the understanding of DSAP's pathophysiology have opened new therapeutic avenues. The disorder has been linked to mutations affecting the mevalonate pathway, a critical metabolic route involved for cholesterol biosynthesis. These mutations result in the accumulation of mevalonate and its metabolites, which are thought to contribute to the inflammatory phenotype characteristic of DSAP. Accordingly, targeting the mevalonate pathway has emerged as a promising therapeutic strategy.^1^, ^3^

One such approach involves the topical application of cholesterol and statins (e.g., lovastatin or simvastatin). This dual action therapy aims to restore cholesterol – an essential component of the skin barrier – while simultaneously inhibiting the accumulation of toxic mevalonate intermediates. This concept was first successfully applied by Paller et al. in 2011 for the treatment of congenital hemidysplasia with ichthyosiform erythroderma and limb defects (CHILD) syndrome, a related disorder involving defects in the same pathway.^4^ In 2019, Atzmony et al. adapted this approach for DSAP, marking a pivotal step in its therapeutic exploration.^5^ Since then, several small case series have reported encouraging outcomes, demonstrating notable reductions in lesion count, erythema, and scaling within a few weeks of application.^6^, ^7^, ^8^, ^9^, ^10^ A recent meta‐analysis encompassing 33 patients found an overall response rate of 93% following twice‐daily application of the formulation for 8 weeks.^11^ However, data on long‐term efficacy and safety of this approach remain limited. Given the chronic nature of DSAP and the paucity of effective treatment alternatives, evaluating long‐term disease control with simvastatin 2%/cholesterol 2% (SC) cream represents an essential next step in addressing this unmet medical need.

## AIM

This investigation aims to explore the efficacy and safety of topical SC cream in a larger cohort of patients over an extended treatment duration. In recognition of the chronic and progressive nature of DSAP, we also seek to develop a structured long‐term management strategy tailored to sustain disease control and symptom stabilization. Furthermore, we propose an optimized therapeutic regimen designed to improve treatment adherence and long‐term outcomes. A key objective of this investigation is to assess whether SC therapy may play a role in preventing malignant transformation, particularly the development of squamous cell carcinoma in individuals with DSAP. To address the currently limited understanding of the disease's overall burden, we additionally evaluated health‐related quality of life (QoL) using the Dermatology Life Quality Index (DLQI), as no such data has been reported to date.

## PATIENTS AND METHODS

All patients who were diagnosed with DSAP at the Department of Dermatology at University Medical Center Mainz between March 2022 and June 2023 were screened for inclusion in this analysis. The follow‐up period extended to 18 months. Treatment was administered as part of routine clinical care, and all patients were informed about the individualized therapeutic approach. The SC cream was prescribed as a compounded formulation, prepared by patients' local pharmacies, using the following composition: simvastatin 2%, cholesterol 2%, preserved water, and *Unguentum Cordes*. The recommended treatment protocol involved twice‐daily application of SC cream to affected areas. Upon confirmation of good tolerability and initial clinical response, application frequency was reduced to once daily (typically after 8–12 weeks). Over time, a proactive maintenance regimen was adopted, consisting of twice‐weekly application, usually within the first 6 months of treatment initiation.

Patients were eligible for inclusion if they returned for at least one follow‐up visit and had photographic documentation available at baseline and at least one subsequent time point. Clinical photographs were obtained at baseline (month 0) and, when feasible, at months 3, 6, 9, 12, 15 and 18 (± 1 month).

Disease severity was assessed by two physicians using a five‐point *Investigator Global Assessment* (IGA) scale based on photographic documentation (0 = no signs or lesions, 1 = slight, 2 = mild, 3 = moderate, 4 = severe, 5 = very severe). All data were anonymized and analyzed following the completion of data collection. Patients completed the DLQI to evaluate health‐related QoL. Additionally, the emergence of new cutaneous tumors and any adverse effects related to treatment were recorded. Demographic data including age at treatment initiation, sex, previous treatments, and history of skin cancer were collected for all patients.

Statistical analyses were conducted using descriptive methods. Absolute and relative frequencies were calculated for categorical variables. Given the low sample size, significance of DLQI scores was analyzed using the paired Wilcoxon test. To assess changes in clinical outcomes over time, Linear Mixed Models (LMMs) were applied using SPSS, accounting for within‐subject correlations. Separate LMMs were conducted for different anatomical sites, with residual maximum likelihood (REML) estimation used to handle missing data under the missing at random (MAR) assumption. Both fixed and random effects were examined to evaluate longitudinal trends.

## RESULTS

### Patient characteristics

A total of 26 patients with DSAP were initiated on treatment with SC cream during the observation period. Of these, 19 patients were included in the final analysis. Seven patients were excluded due to insufficient photographic documentation at the start of therapy, rendering clinical evaluation infeasible. The mean age at treatment initiation was 66.89 years (range: 45–85 years). The majority of patients were female (58%). 14 patients (74%) had received prior treatments for DSAP, 11 (58%) having undergone more than one therapeutic approach. The most frequently reported previous treatments included topical imiquimod (5% or 3.75% cream, 71%), photodynamic therapy (red light and/or [artificial] daylight, 64%), and topical diclofenac‐HA (36%). Other prior treatments encompassed 5‐FU, cryosurgery, ingenol mebutate, alpha hydroxy acid peeling, calcipotriol, and both topical and systemic retinoids, administered in various regimens (Table 1). The longest follow‐up period in this cohort was 18 months (n = 4, 21%). Seven patients (37%) were evaluated after 15 months and eight patients (42%) after 12 months following treatment initiation.

**TABLE 1:** Patient characteristics.

	n = 19
**Age** [years (range)]	66.89 (45‐85)
**Sex** [n (%)]	
Male	8 (42)
Female	11 (58)
**Previous therapies** [n (%)]	
Total	14 (74)
More than one	11 (58)
Imiquimod	10 (71)
PDT	9 (47)
Diclofenac‐HA	5 (36)
**Skin tumors in med history [n (%)]**	
Total	4 (21)
SCC	4 (21)
BCC	4 (21)
Melanoma	1 (5)
AK	8 (42)
**Affected body regions [n (%)]**	
Arm left	16 (84)
Arm right	16 (84)
Leg left	14 (74)
Leg right	14 (74)
Chest	2 (11)
Abdomen	1 (5)

*Abbr*.: AK, actinic keratosis; BCC, basal cell carcinoma; PDT, photodynamic therapy; SCC, squamous cell carcinoma

### Efficacy of SC cream

**FIGURE 1:**
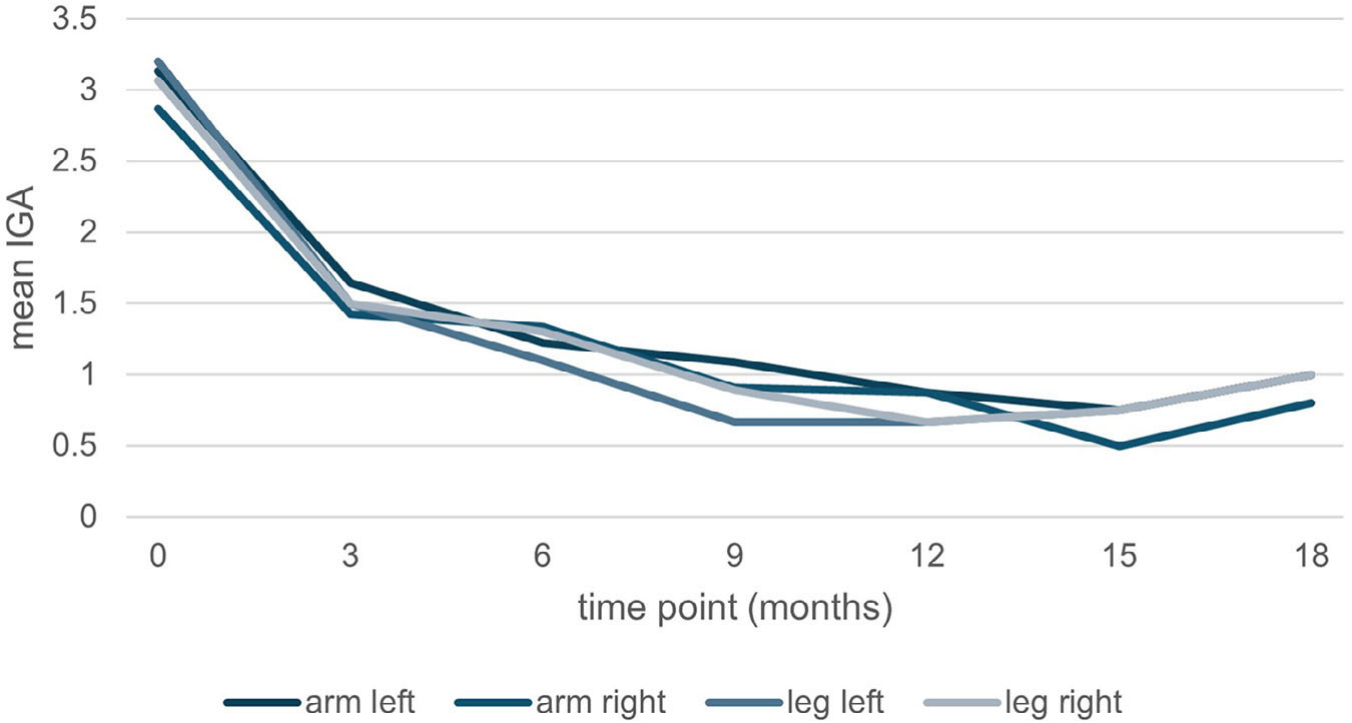
Mean IGA scores for left arm, right arm, left leg, and right leg at different time points of analysis. IGA reduction was significant across all evaluated body regions (p < 0.001).

The most frequently affected body region was the upper extremities (84%) followed by the lower extremities (74%). Lesions were bilateral in all patients. Additionally, two patients (11%) had involvement of the chest, and one patient (5%) presented with lesions on the abdomen (Table 1). Regarding the extend of treatment areas, eight patients applied SC cream to two body regions (42%), one patient to three body regions (5%), nine patients to four body regions (47%) and two patients to five body regions (11%). IGA scores demonstrated a reduction in disease across all patients and all treated body regions (Table 2, Figure 1). A significant decrease in IGA scores was observed over time, with the most pronounced improvement occurring within the first 3 months of treatment. This therapeutic effect was sustained throughout the follow‐up period of up to 18 months. Improvement during the first 6 months was significantly greater than that observed between months 9 and 18. To assess treatment efficacy over time, a LLM was conducted using time (visits: baseline and every 3 months up to 18 months) as fixed effect. Random intercepts for subjects and random slopes for time were included to account for individual variability. An autoregressive covariance structure was used to model the correlation of repeated measures. The main effects of time were statistically significant for all extremities (right arm F[6, 31.949] = 16.361, p < 0.001; left arm F[6, 31.890] = 19.527, p < 0.001;right leg F[6, 31.109] = 27.949, p < 0.001; left leg F[6, 32.211] = 19.828, p < 0.001), indicating a change in IGA over time. Estimated marginal means showed a marked reduction in IGA scores from baseline (right arm: M = 3.133, SE = 0.269; left arm: M = 2.867, SE = 0.247; right leg: M = 3.200, SE 0.231; left leg: M = 3.067, SE = 0.249) to 18 months (right arm: M = 0.748, SE = 0.377; left arm: M = 0.671, SE = 0.335; right leg: M = 0.853, SE 0.337; left leg: M = 0.818, SE = 0.390). Random intercept variances for subjects were 0.649, 0.574, 0.283 and 0.350 for analysis of the right arm, left arm, right leg, and left leg, respectively, indicating low interindividual variability at baseline. The model could not be fitted for the abdomen and chest due to lack of observations. No statistically significant differences in treatment response were observed between arms and legs. For all extremities, mean IGA scores fell below 1 after 9 months of treatment, indicating minimal or no clinical signs of DSAP (Figure 1). The average IGA reduction across all extremities was 1.55 points after 3 months, 1.83 after 6 months and 2.18 after 9 months. Greater variability in IGA scores emerged after 9 months, suggesting increased heterogeneity in response as lesion severity diminished. A complete response defined as IGA 0 in all treated body regions was achieved in three patients (16%). Two of these reached complete remission after 3 months and one after 9 months. An additional ten patients (53%) achieved a best response of IGA 1, indicating only slight residual lesions. Overall, 59% were lesion‐free or only mildly affected following treatment with SC creme. It should be noted that from months 12 onward, the number of available data points declined markedly due to the limited cohort size, which may affect the robustness of analyses at later time points.

**TABLE 2:** Mean Investigator Global Assessment (IGA) scores for each evaluated body region at different time points (months) of analysis.

	Timepoint (m)	0	3	6	9	12	15	18
Arm left	Mean IGA	3.13	1.64	1.22	1.09	0.88	0.75	1.00
	n	15	14	9	11	8	4	5
Arm right	Mean IGA	2.87	1.43	1.33	0.91	0.88	0.50	0.80
	n	15	14	9	11	8	4	5
Leg left	Mean IGA	3.20	1.50	1.10	0.67	0.67	0.75	1.00
	n	15	14	10	9	6	4	5
Leg right	Mean IGA	3.07	1.50	1.30	0.89	0.67	0.75	1.00
	n	15	14	10	9	6	4	4
Chest	Mean IGA	2	1	0	–	1	0.5	0
	n	2	2	1	–	1	2	1
Abdomen	Mean IGA	3	2	–	1	–	–	–
	n	1	1	–	1	–	–	–

*Abbr*.: IGA, Investigator Global Assessment; m, months; n, number of patients

**FIGURE 2:**
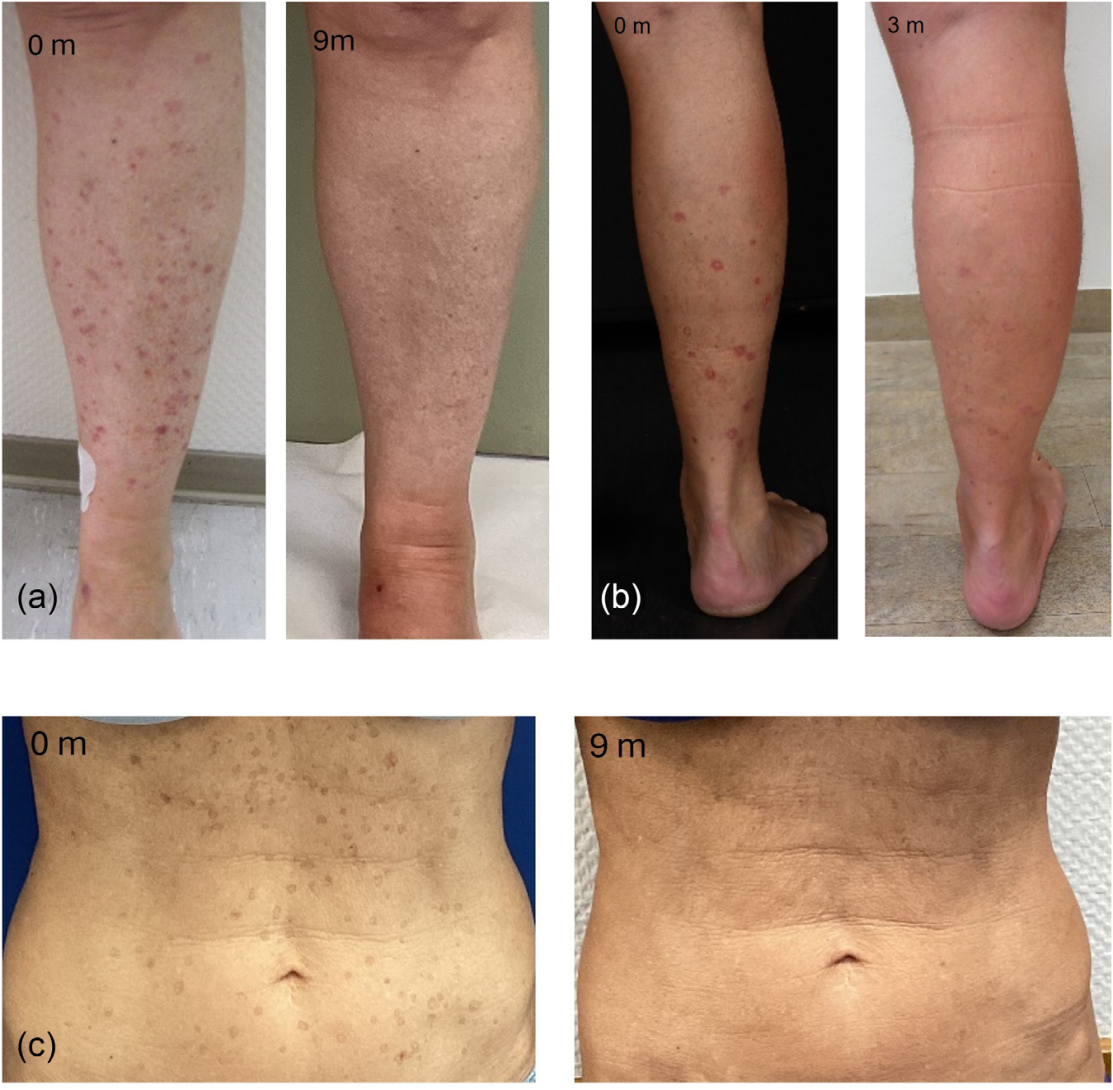
(a) Patient 8, left leg; (b) Patient 10, right leg; (c) Patient 18, abdomen. Time points as indicated in the photographs.

Representative clinical photographs of three patients are provided in Figure 2.

### Patients’ quality of life

**FIGURE 3:**
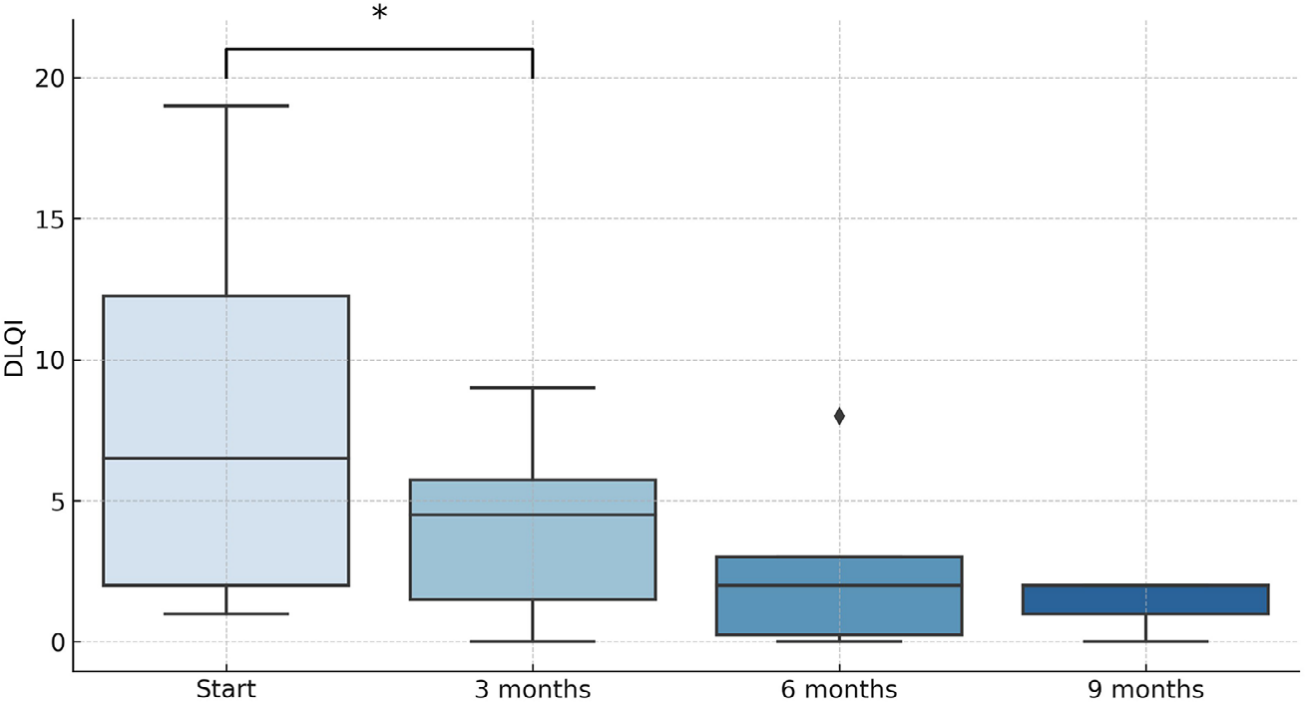
Median Dermatology Life Quality Index (DLQI) scores at the indicated time points. Statistically significant improvement at month 3 compared with baseline (p = 0.027).

The impact of DSAP on patients’ QoL was assessed using the DLQI, which was completed during the in‐house visits prior to physical examination. Due to the real life setting of this work, DLQI data were not systematically collected at each visit. In total, eight patients (42%) completed the DLQI at baseline and at least once during treatment (after 3, 6, and/or 9 months). DLQI scores showed a clear downward trend over time, indicating a continuous improvement in health‐related QoL. At baseline, patients reported a moderate impairment on their QoL (median: 6.5; mean 8.25), with scores ranging from one (no effect on patient`s life) to 19 (very large effect). After 3 months of treatment, the median DLQI score decreased to 4.5 (mild impairment; mean: 3.67), representing a statistically significant improvement compared to baseline (p = 0.027) At 6 months, the median further decreased to 2.0, approaching the threshold for no impairment, showing a trend toward significance (p = 0.094). By month 9, the median remained stable at 2.0, though the difference from baseline was no longer statistically significant (p = 0.25), likely due to the small sample size. The variability in DLQI scores was highest at baseline and declined over time, with score ranges between 0 and 8 at both the three‐ and six‐month time points. Comparisons between later follow‐ups were not statistically significant, suggesting that the most substantial improvement in QoL typically occurs within the first 3 months of treatment (Figure 3).

### Tumor development and adverse events

Given that DSAP is recognized as risk factor for the development of skin cancer, particularly squamous cell carcinoma (transformation risk about 3 to 11%),^1^ we had a special interest in evaluating tumor growth in our patients focusing on the emergence of new lesions during treatment with SC creme. About one fifth of the study population (n = 4, 21%) had a prior medical history of invasive skin cancer, including squamous cell carcinoma (n = 4, 21%), basal cell carcinoma (n = 4, 21%) and malignant melanoma (n = 1, 5%). Above that, dermatopathological records revealed a history of actinic keratosis in eight patients (42%) (Table 1). Importantly, no new malignant skin tumors were detected while patients were undergoing therapy with SC cream during the whole observation period.

Overall, SC cream was well tolerated. Some patients reported dissatisfaction with the formulation's texture, describing it as excessively greasy. Adverse events were reported by three patients (16%), including two cases of eczematous lesions and one case of intermittent pruritus. Notably, all three patients had a known history of atopic dermatitis. Additionally, in one patient a Type IV hypersensitivity reaction was clinically suspected. However, no confirmatory patch testing was performed, and the treatment was discontinued. One patient who was not included in the efficacy analysis due to missing baseline photographic documentation developed a confirmed Type IV allergy to simvastatin that was verified by patch testing. In this case, atorvastatin was tested suitable as an alternative component for formulation.

## DISCUSSION

DSAP is a chronic, therapy‐resistant skin disorder with a considerable unmet medical need. While multiple therapeutic options exist, none have demonstrated consistent efficacy, and many are associated with considerable side effects. Additionally, DSAP is linked to an elevated risk of epithelial skin cancers, particularly squamous cell carcinoma. Given the chronic nature of the disease and its oncogenic potential, effective long‐term treatment strategies are highly desirable.

The increasing understanding of DSAP's molecular pathogenesis, particularly its association with mutations affecting the mevalonate pathway, provides a strong rationale for targeted therapies. Topical formulations combining statins and cholesterol represent a novel therapeutic approach that directly addresses the underlying metabolic dysfunction. While simvastatin was used in our study, previous reports have also demonstrated the feasibility of using lovastatin or atorvastatin in similar compounded formulations, further supporting the adaptability and potential of this approach.^5^, ^7^, ^9^, ^10^

However, existing literature on statin/cholesterol‐based therapies remains limited, with most studies comprising fewer than a dozen patients and/or short follow‐up durations typically spanning only eight to 12 weeks.^5^, ^6^, ^7^, ^8^, ^9^, ^10^ Given the chronic and progressive course of DSAP, long‐term disease control is crucial. Our study contributes to filling this gap by extending the follow‐up period to a maximum of 18 months, providing valuable insights into the sustained efficacy of SC cream.

The findings of our cohort align with previously published literature, as our patient population predominantly comprised middle‐aged individuals (mean age: 66.89), with a slight female predominance and multiple prior treatment failures. The disease primarily affected the extremities in a bilateral distribution, which is consistent with the known characteristics of DSAP. As previously reported by others, our cohort also exhibited a very high therapeutic response to SC cream. A first meta‐analysis on statin therapy for DSAP included 33 patients. The analysis showed that more than 90% of patients experienced an improvement in skin lesions; however, the therapy was consistently administered twice daily, with an average treatment duration of 8 weeks.^11^ Similarly, our study observed significant IGA reductions during the first 3 months. However, the focus of our investigation was on maintaining the therapeutic effect while simultaneously reducing the frequency of application, particularly to ensure long‐term patient compliance and disease control. A key consideration in long‐term topical therapy is patient compliance, particularly when frequent applications are required. To address this, we designed a proactive treatment regimen that reduced application frequency over time, ultimately transitioning to a twice‐weekly maintenance schedule. This approach, inspired by treatment strategies in other chronic inflammatory dermatoses such as atopic dermatitis, may enhance adherence while maintaining disease control.^12^, ^13^, ^14^ Our findings show that most of the therapeutic gains occur within the first 6 months and are maintained with reduced frequency, supporting the effectiveness of this proactive strategy in DSAP.

Interestingly, the treatment response was consistent across all evaluated extremities, with no significant differences in healing trajectories between arms and legs. This finding is notable, as chronic inflammatory skin diseases often show slower resolution on the lower extremities.^15^, ^16^, ^17^ The uniform response pattern in DSAP suggests that anatomical location may play a lesser role in treatment dynamics.

QoL data in DSAP patients remain scarce. Blyth et al. reported a baseline DLQI median of 5 (range 2–21) but did not take any questionnaires during or after the treatment.^6^ Our cohort revealed a median baseline DLQI of 6.5 (range 1–19) indicating moderate impairment with wide interindividual variability – highlighting the heterogeneous impact of the disease. Interestingly, baseline DLQI did not correlate with clinical disease severity assessed via IGA. Due to the real‐world setting of the study, the DLQI was not systematically collected. However, the available data clearly indicates a significant improvement in QoL within the first 3 months, which was sustained for up to one year. Further studies with a larger patient cohort are needed to investigate this important topic in more detail.

SC cream was generally well tolerated. Adverse events were limited to three patients (16%), who experienced eczematous lesions or intermittent pruritus – symptoms potentially attributable to underlying atopic diathesis. In one case, Type IV hypersensitivity was suspected, though not confirmed due to lack of patch testing. Notably, we documented a confirmed case of Type IV allergy to simvastatin in a patient outside this cohort, consistent with a previous report by Ahrens et al.^18^, ^19^ A therapeutic alternative (atorvastatin) was well tolerated, and systemic use of simvastatin remained possible. Whether sensitization is related to simvastatin itself or to excipients used in compounded formulations remains unclear and warrants further investigation, especially considering the widespread off‐label compounding of statin creams from oral formulations.

Our findings support the use of SC cream as a promising personalized and targeted long‐term treatment option for DSAP. Patients demonstrated marked and sustained improvement in lesion severity, regardless of baseline disease activity, with generally good tolerability. Importantly, no new malignant skin tumors were observed during the treatment period. While this does not confirm a protective effect against skin cancer, it raises the possibility that improved disease control may contribute to a reduced risk of malignant transformation. To our knowledge, this is the first study to evaluate long‐term use of SC cream with progressive tapering of application frequency, highlighting its potential as a viable maintenance strategy in chronic DSAP.

### Limitations

Several limitations should be acknowledged when interpreting our findings. This investigation was conducted in a real‐world clinical setting rather than as a controlled interventional trial, leading to variability in data collection and follow‐up intervals. Furthermore, treatment adherence was not systematically monitored, limiting our ability to assess the compliance objectively. Although our follow‐up period of up to 18 months exceeds that of most prior studies, it remains insufficient to draw firm conclusions regarding the potential of SC cream to prevent malignant transformation. Further longitudinal studies with standardized follow‐up and larger patient cohorts are necessary to validate and expand upon these preliminary findings.

## CONCLUSION

Topical SC cream represents a significant step toward a targeted, individualized, and patient‐centered approach to the management of DSAP. This study demonstrates its potential to effectively control disease activity over an extended period, with a favorable safety and tolerability profile. Future research should aim to optimize formulation components, evaluate long‐term outcomes more comprehensively, and investigate the potential role of mevalonate pathway inhibition in reducing skin cancer risk among DSAP patients. Additionally, the development of standardized, commercially available preparations – and the exploration of systemic administration routes – may further enhance treatment accessibility and efficacy. Despite the need for continued research, SC cream emerges as a promising therapeutic option for sustainable long‐term disease control in DSAP.

## CONFLICT OF INTEREST STATEMENT

None.
